# The epithelial to mesenchymal transition (EMT) and cancer stem cells: implication for treatment resistance in pancreatic cancer

**DOI:** 10.1186/s12943-017-0624-9

**Published:** 2017-02-28

**Authors:** Pingting Zhou, Bo Li, Furao Liu, Meichao Zhang, Qian Wang, Yuanhua Liu, Yuan Yao, Dong Li

**Affiliations:** 1 Department of Oncology, Shanghai Ninth People’s Hospital, Shanghai Jiaotong University School of Medicine, Shanghai, China; 2 Department of Bone Tumor Surgery, Changzheng Hospital, Second Military Medical University, Shanghai, China; 3 Department of Chemotherapy, Nanjing Medical University Affiliated Cancer Hospital, Cancer Institute of Jiangsu Province, Nanjing, Jiangsu China; 4 Department of Radiation Oncology, Shanghai Ninth People’s Hospital, Shanghai Jiaotong University School of Medicine, Shanghai, China

**Keywords:** Pancreatic cancer, Cancer stem cell, Epithelial-to-mesenchymal transition, Resistance

## Abstract

The mechanical properties of epithelial to mesenchymal transition (EMT) and a pancreatic cancer subpopulation with stem cell properties have been increasingly recognized as potent modulators of the effective of therapy. In particular, pancreatic cancer stem cells (PCSCs) are functionally important during tumor relapse and therapy resistance. In this review we have surveyed recent advances in the role of EMT and PCSCs in tumor progression, metastasis and treatment resistance, and the mechanisms of integrated with biochemical signals and the underlying pathways involved in treatment resistance of pancreatic cancer. These findings highlight the importance of confirming stem-cells markers and complex molecular signaling pathways controlling EMT and cancer stem cells in pancreatic cancer during tumor formation, progression, and response to therapy.

## Background

Pancreatic cancer (PC) is one of the poorest prognosis malignancies with a 5-year survival rate of less than 5% and a median survival of no more than 6 months after diagnosis [1, 2]. Even among patients diagnosed with early-stage disease who undergo clean surgical margins resection (R0 resection) followed by adjuvant chemotherapy, the median survival rate is approximately 2 years, with a 5-year survival of 15–20% [3–5]. This devastating situation is due to several factors. First, due to the absence of effective tools for an early detection, most patients at the time of diagnose have locally advanced or metastatic disease, and lose the opportunity of surgical resection. Second, even for those patients who undergo surgical resection, the prognosis is poor due to early relapse and distant metastasis. Metastasis is a characteristic of pancreatic cancer and the leading cause of mortality among cancer patients [6]. Finally, PC shows profound resistance to relative chemotherapy and radiation treatment. Cancer cells resistant to treatment usually show more aggressive, such as accelerated metastasis to distant organs and tissues. Thus treatment resistance becomes the major challenge in clinical cancer therapies. The focus on the management of PC patients, especially those in advanced stages, is to understand the pathophysiological mechanisms of therapy resistance and overcome the resistance.

Cellular heterogeneity is a well-recognized property of both normal and malignant tissues. The difference is that heterogeneity in the normal tissues is an ordered developmental program. However, tumors are composed of a small set of distinct cells termed cancer stem cells (CSCs), which is capable of driving tumor initiation and development. The CSCs model, on the other hand, suggests that the biology process of the tumor is driven by a small population of cells with the stem cell properties of sustaining growth and an ability to differentiate into the entire heterogeneous tumor [7]. Dick and colleagues in 1997 identified the first cancer stem cell in hematopoietic malignancies, such as acute myelogenous leukemia and chronic myelogenous leukemia using cell surface marker expression [8, 9]. Hematopoietic stem cells (HSCs) can self-renew and differentiate into all the cells of the hematopoietic system, and are responsible for lifelong blood production [10]. After the discovery of CSCs in leukemias, the first CSCs in solid tumors were identified in breast tumors [11], leading to much research in a variety of tumors, including glioblastoma [12], pancreas [13, 14], melanoma [15], prostate [16] and colon [17]. PCSCs have been first discovered in 2007 and since then have conducted as a subpopulation of cancer cells with special functional features including self-renewal and exclusive in vivo tumorigenicity. Furthermore, the resistance of PC to standard chemotherapy and radiation treatment may in part be due to the existence of CSCs, which can express multidrug-resistant membrane transporters, aberrantly activate proliferation signaling pathways and increase the capability of repairing DNA.

Although there are a growing number of studies that support the CSCs model in cancer, diverging theories exist on the precise origin of cancer stem cells. It is not yet known whether they originate from the tissue’s normal stem cells by the accumulation mutations or the acquisition of the mutations in more-differentiated cells. Recent studies have implicated that the process termed epithelial-to-mesenchymal transition (EMT) is associated with features of CSCs [18, 19]. This review focuses on recent research findings related the role of EMT and CSCs on chemotherapy and radiotherapy resistance in pancreatic cancer, helping understand the complex biology of treatment resistance for the more effective treatments for PC patients.

### EMT in cancer

In addition to the field of EMT in normal embryonic development, there are numbers of new work on the role of EMT in tissue fibrosis and cancer metastasis [20–22]. In March 2008, at a Cold Spring Harbor Laboratory meeting about EMT, the scientists classified EMT into three general subtypes based simply on the different functional consequences [23]. Type 1 EMT can generate mesenchymal cells (primary mesenchyme) that have the potential to generate secondary epithelia by mesenchymal-epithelial transition (MET), which is associated with embryonic gastrulation and neuroepithelial giving rise to monile neural crest cells. Type 2 EMT is associated with wound healing, tissue regeneration, and organ fibrosis, which are in essence an unabated form of wound healing in response to persistent inflammation. Type 3 EMTs occur in epithelial neoplastic cells undergoing genetic and epigenetic changes, producing outcomes far from those observed in other two types EMT. Neoplastic cells undergoing type 3 EMT may migrate through the blood stream and generate secondary nodules, threatening manifestations of cancer progression. It is now widely accepted that in epithelial cancers, including pancreatic cancer, EMT is associated with the three major steps of cancer development: invasion, dissemination and metastasis [24, 25]. While the reverse process, a mesenchymal to epithelial transition (MET), is believed to support metastatic progress once migratory cells have reached their destination [26, 27]. During EMT, cells lose their epithelial cell-cell adherens junction and apical-basal cell polarity and acquire mesenchymal characteristics with spindle-like cell shape and with the ability to migrate [28].

A variety of markers have been used to demonstrate EMT. E-cadherin, intergrins, and cytokeratins are the most commonly used epithelial markers and N-cadherin, vimentin or fibronectin for the mesenchymal [26]. In recent years, the cadherin switch from E-cadherin to N-cadherin, have been increasingly used to monitor EMT during cancer development. However, cells do not gain mesenchymal traits in a partial EMT. Early EMT might only involve loss of E-cadherin and do not gain N-cadherin, this phenomenon may lead to different biological results of the intermediate states and likely behave distinctly during migration and invasion to those that do gain N-cadherin [29, 30]. Hence, it is important to consider not only epithelial or mesenchymal traits during EMT but also other processes related to EMT, such as invasion, increased survival or decreased proliferation.

EMT change is triggered in a number of distinct molecular processes including the expression of specific cell-surface proteins and the activation of transcription factors (TFs). The list of potent EMT-inducing transcription factors (EMT-TFs) has been growing ever since [31], EMT-TFs belong to different families, including superfamily, SNAI1 (previously known as Snail) and SNAI2 (previously known as Slug), two ZEB factors, ZEB1 (also known as dEF1/TCF8) and ZEB2and Twist. Other TFs have been shown to be related with EMT, including Prrx1,Sox4 and Sox9 [32, 33], Klf4 [34] and members of the AP-1 (Jun/Fos) family [35]. It is reported that EMT-TFs have been involved not only in migration and invasion but also in the protection from senescence and apoptosis, regulation of cell progression and resistance to chemotherapy and radiotherapy [36–38]. These EMT regulators have been shown to repress the expression of E-cadherin [39–42] through binding to conserved E-box sequences (mainly of the CAGGTG type) in the promoter of E-cadherin [41, 43]. Loss of E-cadherin can drive certain epithelial cells toward a mesenchymal state [44].

EMT associated with tumors can be induced by various secreted factors from the stroma, such as transforming growth factor beta (TGF-β), hepatocyte growth factor (HGF) and platelet-derived growth factor (PDGF). Among these, TGF-β has received substantial attention as a major inducer of EMT during embryogenesis development, cancer progression and fibrosis. Consequently, TGF-β-induced EMT has been better understood than EMT in response to other inducers. In response to TGF-β-ligand binding, TGF-β receptors I and II (TGF-βRI/ TGF-βRII) which have a serine/threonine kinase activity lead to phosphorylation of Smad2 and Smad3, members of the Smad protein family [45]. After the phosphorylation, a Smad signaling cascade is activated, resulting in nuclear translocation of Smad4, which drives a wide range of tumor-promoting factor genes transcription [46]. TGF-β additionally induces Smad-independent signaling. Among the non-Smad signaling responses, TGF-β can regulate EMT through the activation of Rho GTPases, MAP kinase (MAPK) pathways and the PI3 kinase-Akt-mTOR pathway [47, 48]. On the other hand, non-Smad signaling can impact the activation of Smad signaling in TGF-β induced EMT. For example, Akt activation can sequester Smad3 through unphosphorylated Smad3, impacts Smad activation in response to TGF-β in EMT [49]. P38 MAPK cooperates with Smad3/4 in TGF-β associated EMT through the transcription factor ATF2 [50]. TGF-β can interact with other growth factors such as the epidermal growth factor (EGF) to influence the malignant transformation of CSCs as well as the activation of cancer-associated stromal fibrosis [51–58].

**Fig. 1:**
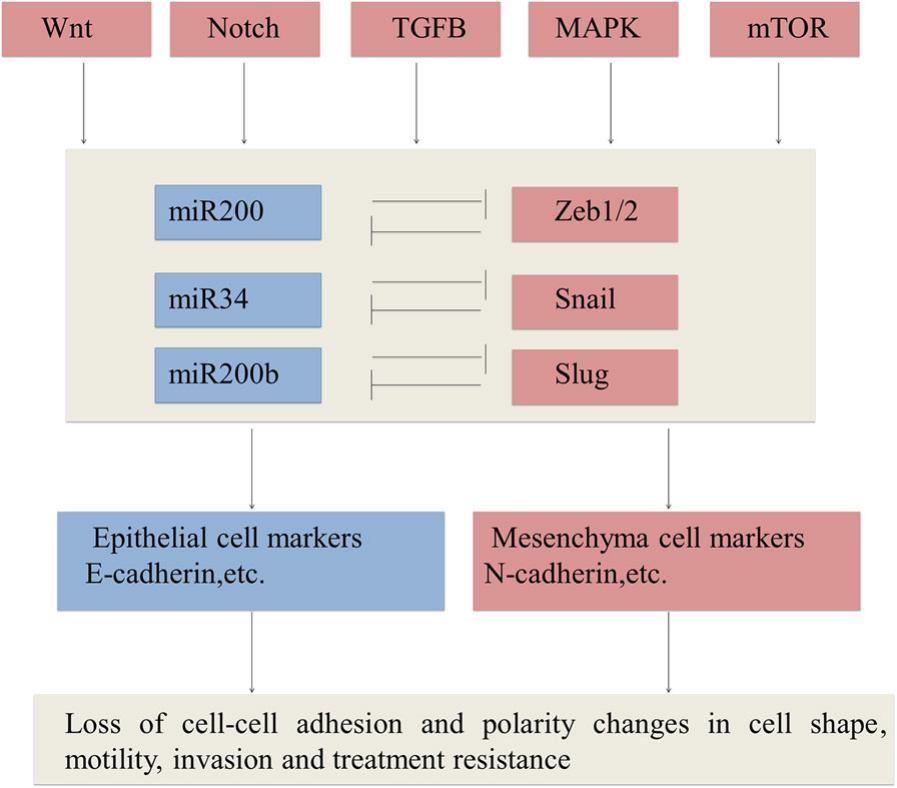
The Core Regulatory Machinery of EMT. Tumorigenesis activate EMT-promoting transcription factors of the TWIST, SNAIL and ZEB families through pathways known to play critical roles in both embryogenesis and tumour development, including the WNT, NOTCH, TGF-β, RAS and NF-κB cascades. MicroRNAs suppress production of these transcription factors as well as multiple markers defining the epithelial or mesenchymal characteristics. These microRNAs can therefore promote EMT (blue) or repress EMT and enhance MET programs (orange)

While transcriptional regulation of EMT has been extensively studied, post-transcriptional, translational and post-translational regulators are recently appreciated in several studies [59, 60]. The number of miRNAs that has been reported to be associated with EMT and MET is becoming as extensive as the list of EMT-TFs [61]. There is no doubt that one of the most concern is the miR-200 family, which has five members classified in to two groups; miR-200a, miR-200b and miR-429 on human chromosome 1, miR-200c and miR-141 on human chromosome 12 [62]. Their low expression of individual family members results in EMT progression by enhancing levels of EMT-TFs Zeb1 and Zeb2, which blocks EMT or induces MET [63]. Conversely, Zeb1/2 directly binding to miR-200 promoters can repress the expression of miRNAs. This interaction between Zeb1/2 and miR-200 family members not only determines cell morphology but also controls cell migration and invasion [64, 65]. Reversely, microRNA array analyses have suggested that members of miR-200 family were markedly downregulated in TGF-β–induced EMT and in cancer cell lines displaying an EMT phenotype [63, 66]. In addition to the members of the miR200 family, miR-10b, miR-373, and miR-520c also play roles in the progression of cancer [67, 68]. It`s reported that miR-21 is enhanced during the process of TGFβ-induced EMT [69]. The details of the interaction of all these factors with each other are stated in Fig. 1.

### Pancreatic cancer stem cells

**Fig. 2:**
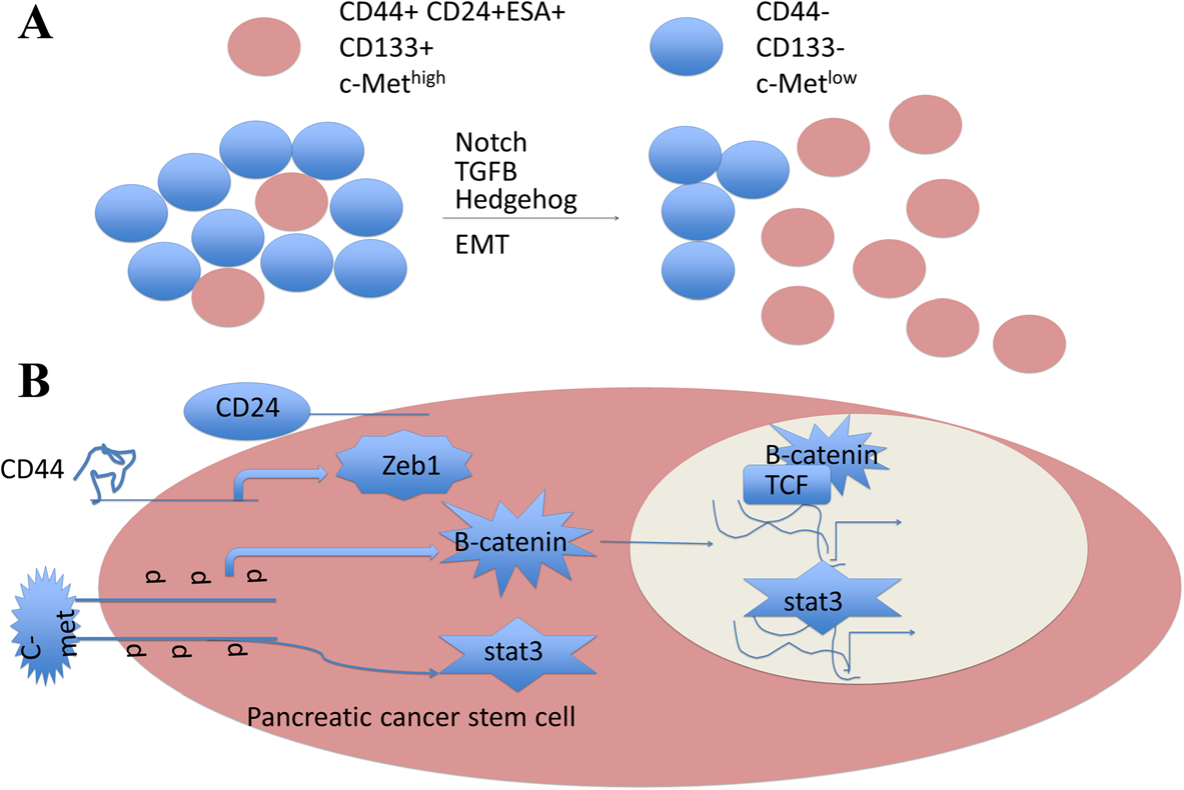
Contribution of EMT and related signaling to PCSCs. **a** PCSCs with tumor-initiating capability can be identified by the expression of a distinct set of marker proteins, such as CD44, CD24, CD133 or c-Met. These CSCs can self-renew and differentiate into a number of cell types to generate the heterogeneity of the originating tumor. Inducers of EMT such as TGF-β, HH or Notch cause cells to acquire a CD44+ CD24 + ESA+ phenotype, reminiscent of PCSCs. **b** The PCSC cell surface markers CD24, and CD44 likely promote cell–cell interactions, the c-Met respond to secreted ligands to active developmental pathways, such as β-catenin, Notch and Stat3 in PCSCs. These pathways stimulate the expression of genes that regulate stem-cell properties, such as self-renewal

The hypothesis of PCSCs has been hotly controversial for many years. A new understanding of PC progression and recurrence has led to the theory that tumorigenesis was facilitated by a distinct population of cancer cells with the properties of stem cells (Fig. 2) [70]. The presence of CSCs has been studied in a variety of hematopoietic and solid organ malignancies [8, 11]. The current consensus agreement describes that CSCs identified based on specific cell-surface markers are able to self-renew and differentiate into the heterogeneous of cancer cells that comprise the tumor [71]. Opinions on the precise origin of cancer stem cells existed different. One theory is that CSCs originate from the accumulation mutations occurring in normal stem cells and these mutations ultimately trigger a malignant transformation. Alternatively, some studies show that mutations in more-differentiated cells may develop the capacity for unregulated self-renewal and with stem cell-like properties [72, 73]. Hence, the term CSCs, does not indicate the origin of cancer cells, but to the cells that maintain the tumorigenesis [71, 74].

In 1997, the first CSC was isolated in myeloid leukemia using cell surface marker expression [8]. Isolating CSCs in solid organs was based on studies in hematopoietic malignancies. However, cell surface markers used to identify CSCs in epithelial solid organ were distinct from those of leukemia CSCs. In PC, CSCs have initially been identified by being characterized as CD44^+^CD24^+^ESA^+^cells and having the ability to form tumors at a much higher frequency than the bulk tumor [13]. The CD44^+^CD24^+^ESA^+^ cells as a reflection of stemness is generally confirmed with the fact that cells enriched for CD44^+^CD24^+^ESA^+^ were far more tumorigenical than other tumor cells. Half of the mice injected with 100 CD44^+^CD24^+^ESA^+^ cells formed tumors, compared with 10000 triple-negative cells, which only 1 in 12 mice developed a tumor. In addition, the tumors formed by the CD44^+^CD24^+^ESA^+^ cells had morphological features similar to those of the original pancreatic ductal adenocarcinoma from patients. These supporting data pointed that a population of cells are responsible for tumor initiation and self-renewal.

In another group, CD133 was used to identify CSCs from human primary PC samples and PC cell lines [14]. Like CD44^+^CD24^+^ESA^+^ cells, 500 CD133^+^ PC cells generated visible tumors that histologically indistinguishable from the primary tumor. In contrast, 10^6^ CD133^−^ PC cells failed to induce tumor formation. By flow cytometry analysis, they showed that CD133^+^ cells and CD44^+^CD24^+^ESA^+^ cells are not identical but have a 14% overlap. Further experiments will need to estimate the tumorigenic potential of this quadruple-positive subset. This study also investigated the role of pancreatic CSCs in metastasis and the results revealed that a subpopulation of CD133^+^ cells that co-express CXCR4 determined the metastatic ability of PC cells. CXCR4 is a chemokine receptor of the ligand stromal cell-derived factor 1 (SDF1), which is reported to be a mediator of tumor invasion and metastasis [75]. Hermann et al. showed that two groups of mice injected CD133^+^ CXCR4^+^ and CD133^+^ CXCR4^−^ cells appeared similar tumor development in the early stage, but followed with liver metastases or no any trace of metastases 2 weeks later in CXCR4^+^ group and CXCR4^−^group respectively [14]. The difference in cell populations was activation of CXCR4. These cells, CD133^+^CXCR4^+^, were noted to have increased in vitro migratory ability and were able to produce metastasis, however CD133^+^CXCR4^−^ did not produce distant metastases. Importantly, CXCR4 inhibition prevented tumor metastasis in mice. These findings may have clinical implications that CXCR4 might be a potential target for therapies to inhibit metastasis of PCSCs.

After the identification of CD44^+^CD24^+^ESA^+^ cells and CD133^+^ cells as a population of PCSCs, Li et al. identified c-Met as a hunman pancreatic CSC marker [76]. c-Met, a member of the receptor tyrosine kinase for hepatocyte growth factor (HGF), has an important role in both normal and malignant development [77]. It was shown to be related with tumorigenesis and drug resistance through activating mutations of pathway components or HGF-dependent autocrine/paracrine in tumor development [78, 79]. They found that c-Met high cells had enhanced tumorigenic potential, whereas c-Met-negative cells did not. They compared the tumorigenicity of different sets of primary tumor cells and identified that c-Met high cancer cell population had the highest tumorigenic potential, compared with pancreatic cancer cells expressing CD44, CD24, ESA and CD133 [76]. Cabozantinib, the c-Met inhibitor, significantly inhibited tumor sphere formation, reduced the population of PCSCs and slowed tumor growth. Additional studies have shown that ALDH1 (aldehyde dehydrogenase 1) and Dclk1 (doublecortin and Ca2/ calmodulin-dependent kinase-like 1) high cells have enhanced sphere-forming ability, suggesting a propensity to be associated with PCSCs function [80].

Several markers have been defined to discriminate PCSCs, but the signaling pathways that regulate these PCSC functions have not been fully studied. Notch signaling is an important regulator of self-renewal and differentiation in the normal and development pancreas, but the dysregulation of the Notch pathway will lead to uncontrolled self-renewal of CSCs [81–84]. In PC, inhibition of the Notch pathway by either knockdown of the Notch ligand Jagged-1 or blocking Notch pathway with inhibitor MRK-003 leads to reduction of tumor sphere formation [85, 86]. On the other hand, the activation of Notch pathway acts to maintain the pancreatic precursor state. These findings indicate that Notch signaling is needed for PCSC function [87]. Nodal and Activin belong to the TGF-β superfamily and they were shown to be important for human embryonic stem cell maintenance [88, 89]. In pancreatic cancer, Nodal/Activin is strongly expressed in PCSCs. The Nodal/Activin pathway can affect the self-renewal capacity and stemness properties of pancreatic CSCs and promote invasion of PCSCs [90]. Several studies have identified other developmental pathways such as mTOR, hedgehog for targeting PCSCs [91, 92]. Compared with normal pancreatic epithelial cells, the expression of HH transcript was increased 46-fold in CD44^+^CD24^+^ESA^+^ cells, which suggests that hedgehog signaling ligand is significantly expressed in pancreatic CSCs [93].

### The relationship between EMT and PCSCs

Of fundamental importance biologically, the activation of EMT process has been associated with the properties of stem cell traits for both normal and neoplastic cells [18, 19]. The biologic link between EMT phenotypes and CSCs has recently been studied in many types of cancer including PC (Table 1) [52, 94–99]. Cells with an EMT phenotype effect molecular characteristics of CSCs; CSCs also express an EMT phenotype. This phenomenon indicated that EMT and CSCs are closely related [100–102]. In breast cancer, Mani and colleagues reported that the overexpression of Twist, Snail or FOXC2 not only made the breast cancer cells with more mesenchymal properties, but also with an increased expression of CD44^+^/CD24^-/low^ breast CSC markers and an increased mammosphere forming efficiency [18, 103]. The similar results also showed in prostate cancer. Prostate cancer cells with an EMT phenotype have increased expression of Sox2, Nanog, Pou5F1, lin28B and/or Notch1 and an enhanced sphere-forming ability [104].

**Table 1:** Principal data regarding the relationship of EMT with PCSCs

Experimental approach	Molecular characteristics of PCSCs	References
Short hairpin RNA (shRNA)-mediated *ZEB1*-knockdown in the two cells with the highest levels of ZEB1(Panc–1 and MiaPaCa–2)	Reduction of CD24+/CD44+ subpopulation, reduced sphere formation in the two cancer cell lines and sphere numbers in subsequent generations decreased expression of stem cell factors such as Sox2, Bmi1 and p63	[96]
CD133 overexpression in Mia PaCa-2 cell	Increased mRNA expression of several EMT-associated genes: SNAI1, ZEB1, Vimentin, CDH2 and MMP9. CD133hi-MIA cells show a more fibroblast-like morphology	[97]
Silenced Snail in Panc-1 cells	A significant decrease in the ALDH^high^ population, reduction initial formation of spheres and sphere numbers in subsequent generations.	[98]
Nestin shRNA in PANC-1 cell and nestin-overexpressing in MiaPaCa-2 cell	Expression of mesenchymal markers, acquisition of invasive properties and high motility/opposite effects	[99]
Isolate the SP cell fraction (side population, a cancer stem cell enriched fraction from Panc-1,KP-1NL and Capan-2 cell lines), incubate SP cells in the presence of TGF-β	Production of cells with mesenchymal-like morphology,alteration such as reduction of E- cadherin mRNA and induction of Snail mRNA and (MMP)-2 mRNA	[52]

ZEB1 (zinc finger E-box binding homeobox 1) is a crucial promoter of EMT. ZEB1 can repress the expression of the miR-200 family and stemness-inhibiting miR-203, resulting in activation of EMT and tumor-initiating capacity in pancreatic cancer [96]. In addition, miR-200 family can suppress the expression of stem cell factors, such as Sox2 and Klf4. It is also suggested that ZEB1and PCSCs marker CD44 regulate each other. ZEB1 enforces CD44 isoforms (CD44s) splicing by repression of epithelial splicing regulator ESRP1 in pancreatic cancer. CD44s, in turn, increases the expression of ZEB1, resulting in a self-sustaining ZEB1 and CD44s expression. The relationship of this novel CD44s-ZEB1 impacts on cancer cell ability, including increased tumorsphere initiation capacity and tumor metastasis [105]. These results suggested that ZEB1 linked EMT and stemness-maintenance in PC.

Nestin was first recognized as a functional stem cell marker in embryonic and adult central nervous system (CNS) stem cells [106]. In addition, recent studies have identified nestin as a CSC marker in brain tumors, ovarian, head and neck, prostate, and PC [107–111]. Compared with parental cells, PC cells with Nestin knockdown exhibited decreased sphere formation and regulated EMT by decreasing slug expression [112]. Overexpression of nestin induced TGF-ß1 and the expression of its receptors through the Smad4-dependent pathway in PC and nestin overexpression induces the EMT of PDAC cells. Meanwhile the excessive TGF- β1 cytokine as a major EMT-inducing soluble factor results in increased nestin expression. Thus, nestin-positive cells apparently use an autocrine positive feedback loop to regulate EMT in PDAC through TGF-β/Smad pathway [99]. In addition, Hypoxia induction increased expression of the PCSCs markers Notch1, Notch4, c-Met, CD133 and the embryonic stem cell markers Nanog, SOX2, FOXA2, SOX17 and PDX-1 [113]. Among above markers, the upregulation of FOXA2 was accompanied by an EMT, with down-regulation of E-cadherin and upregulation of mesenchyme markers Vimentin, Slug, Snail and Twist2. Previous experience in breast cancer showed that Twist2 overexpression not only promoted EMT signaling, but also enhanced colony-forming abilities of stem cells, which suggested that Twist2 may be a master inducer of both EMT and CSC features.

### PCSCs, EMT and treatment resistance

In the clinic, the combination of radio- and chemotherapy with or without surgical intervention is the standard of care in many cancers. Although technical advances in radiation and chemotherapy have improved local control and patient survival. Cancer treatment resistance, including chemoresistance and radioresistance, is still a major challenge in cancer research and treatment [114, 115]. Treatment resistance has become a key obstacle in improving the effectiveness of tumor therapy, resulting in the high mortality in patients diagnosed with PC. This disappointing situation strongly needs to improve on understanding the mechanisms of the treatment resistance, leading to find out novel therapeutic strategies for overcoming the resistance and increasing the survival rate. Potential mechanisms of resistance to antiangiogenic therapy may result from the selection effect directly and indirectly on advantaged subpopulations of tumor and tumor- associated cells [116].

Accumulating researches clearly suggest that CSCs and EMT-type cells play important roles in chemoresistance and radioresistance (Table 2) [117–122]. The role of CSCs contributing to treatment resistance has been reported to be closely related with activation of the DNA damage checkpoint repair, thereby protecting cells from DNA damage and activating the cell survival signaling pathways [114, 115, 123]. Few of the current therapies can eliminate CSCs because they have critical roles in treatment resistance, which might interpret why cancer is difficultly eradicated completely.

**Table 2:** Principal data regarding the role of PCSCs in the induction of treatment resistance

Experimental approach	Molecular characteristics of PCSCs	References
Isolated SP(side population) cell fractions in L3.6pl cell‚ gemcitabine- and 5-FU-resistant L3.6pl cells were established	Induce faster and more aggressive orthotopic tumor growth with higher rates of metastases‚ gemcitabine resulted in an increase of CD24 positive cells and the percentage of SP cells	[117]
Incubated in the presence of 5- fluorouracil (5-FU) for 24 h, and further incubated without 5-FU for 28 days to eliminate 5-FU-sensitive cells.	Certain stemness-genes such as OCT4 and NANOG were enhanced and spheres arose	[118]
Treat Capan-1 and Panc-1 cells with serial concentrations of gemcitabine and counting surviving cells after 6 days,	Stem markers CD44,CD24,CD133,EpCAM,Oct4 and PDX1 increased	[119]
Xenograft tumours were dissociated into single cells and identified SP cells using FACS analysis	SP displayed higher sphere-forming capacity, epithelial-mesenchymal transition and gemcitabine reisistant	[120]
Konckdown of ALDH1 in MIA PaCa-2 cell	The IC50 of gemcitabine decreased, induction of apoptosis and S-phase arrest by gemcitabine.	[121]
Enriched pancreatic cancer stem CD44+/CD24+ cells in PANC-1 cells under sphere forming conditions	Increased resistance to gemcitabine, migration ability, exhibit Epithelial to Mesenchymal Transition (EMT)	[122]

In hematopoietic malignancies, Michor and colleagues identified that a subpopulation of human leukemia stem cells showed resistant to the Abl tyrosine kinase inhibitor imatinib, an effective drug against differentiated leukemic cells. The survived leukemic stem cells regenerated the tumor, providing further evidence supporting the important role of CSCs in tumor relapse [124]. Evidence of the breast CSCs resistance is supported by a study in which tumor biopsies taken from patients with breast cancer during the 12-week chemotherapy treatment conducted increased CSC markers (CD44^+^/CD24^-/low^ and MFSE) [125]. The glioblastoma CSCs were also shown to be responsible to standard therapies resistance in brain tumor. It was identified that the CSC population expressing CD 133 was increased two to four folds in both primary tumors and xenografts after radiation, maybe due to a preferential activation of the DNA damage response [126].

Recently, some studies suggest that pancreatic CSCs may also be resistant to chemotherapy and radiation therapy. With regard to this, Hermann et al. have conducted that human CD133^+^ pancreatic CSCs isolated from pancreatic tumor are highly resistant to standard gemcitabine therapy, which is conventional chemotherapeutic agent against PC [14]. Gemcitabine-resistant cells, which have stronger sphere-forming ability and are more tumorigenic than gemcitabine-sensitive cells in vitro and vivo, were equipped with the similar properties as pancreatic CSCs [127]. Furthermore, it is reported that although the cytotoxic agent gemcitabine can arrest proliferating of CD133 CSCs, the apoptosis of CSCs was not affected, leading to CSCs` returning to stem cell pool when gemcitabine withdrawn. On the contrary, the more differentiated cells (CD133^−^), the vast majority of the tumour cell, became apoptotic under the management of gemcitabine. It is obvious that only the more differentiated tumour cells can be targeted with standard therapy, leaving undifferentiated cancer stem cells resistant to therapy. These results suggest that therapeutic targeting of the activation of apoptosis might provide a tool to sensitize CSCs to therapy.

Tumor-infiltrating immune cells are feature of most solid tumors, can promote chemoresistance and metastasis in aggressive tumors, leading to awful clinical outcomes of cancer patients [6, 128]. It has been illustrated that tumor-infiltrating macrophages (TAMs) can directly induce PCSCs properties through the activation of STAT3 pathway. Conversely, STAT3^+^ CSCs enhance TAM-mediated immunosuppression. Furthermore, targeting TAMs by inhibiting either CSF1 receptor (CSF1R) or chemokine (C-C motif) receptor 2 (CCR2) decreases the numbers of pancreatic CSCs and improves chemotherapeutic efficacy in vivo. The PCSCs response efficiency to chemotherapeutic is highly related with TAMs [129].

The self-renewal potential and resistance to traditional treatment suggest that maybe strategies targeting CSC will improve clinical outcomes. At first, surface proteins used to enrich for pancreatic CSCs maybe good targets for treatment of pancreatic CSCs. For example, inhibiting c-Met with XL18427 or Alk-4 and −7 with SB431542 can eliminate the CSCs in tumors and enhance the antitumor effect of gemcitabine, reducing tumor burden in mice [90]. In addition to reagents targeting surface proteins of PCSCs, several cellular signaling pathways regulating the self-renewal and proliferation ability of PCSCs may be a potential targets against CSCs. Feldmann and colleagues showed that inhibition of Hedgehog pathway reduces a process of PC metastases, which has been linked to the invasion of CSCs [130]. However, Mueller et al. have shown that neither hh inhibition alone nor rapamycin alone or as supplements to chemotherapy can effectively diminish the CSC pool [131]. Chemotherapy, only with the combined inhibition of hh and mTOR (mammalian target of rapamycin) pathway played a role in reducing the number of CSCs to virtually undetectable levels in vitro and in vivo and significantly prolonged survival of mice. However, CD24, CD44, ESA, CD133 and CXCR4 are also expressed on normal stem cells, which share many pathways with CSCs. Consequently, these reagents might lack important specificity in targeting CSCs. It is important to find out effective targeting strategies achieving the aim of tackling CSCs without harming normal stem cells. Besides, the enrichment of the CD44^+^CD24^+^ESA^+^ cells was observed after the ionizing radiation treatment in human primary pancreatic cancer xenografts. CD44 is required for post-radiation recurrence of xenograft tumors in mice. Antibody against CD44 eliminated bulk tumor cells as well as TICs from the tumors. Strategies to target CD44 might be developed to block post-radiotherapy recurrence in patients [132].

## Conclusion

Since cancers are heterogeneous, future novel treatment targets aimed at increasing patient survival will undoubtedly need to consider the heterogeneity of cancer cells. Heterogeneity among cancer cells within the same tumor arises from a consequence of environmental differences, genetic mutation, and reversible changes in cellular properties. Among them one origin of such heterogeneity is EMT and the existence of dedifferentiated cells with CSC-like properties. A better understanding of the properties of EMT and CSCs in PC will play an important role in developing emerging and effective therapies targeting not only the bulk tumor but also the residual cluster of cells that are responsible for the relapse, metastasis and treatment resistance of the tumor. CSC properties have been put forward to explain diverse unsolved clinical problems. However, difficulties confirming solid CSC markers in order to isolate PCSCs have hindered the research identifying their existence in some cancers and studying their biology in clinical applications.

It is now widely accepted that the presence of EMT and a PC subpopulation, with stem cell properties, play important roles in escaping from current clinical specific therapies. In this review we have discussed the detailed process and complex molecular signaling pathways controlling EMT and CSCs in PC during tumor formation, progression, and response to therapy. The combined use of different gene products altered in EMT and PCSCs may represent potential strategies for improving the effectiveness of the diagnostic/prognostic methods and treatments efficacy for cancer patients in the clinics. Targeting CSCs via modification of the Wnt, HH and Notch signaling pathways of these cells holds the promise of preventing treatment resistance. However, additional studies are required to further confirm stem-cells markers and abnormal signaling pathways in CSCs, which have significant correlation with the high treatment resistance of PC.
